# Changes in Body Fat and Related Biochemical Parameters Associated With Atypical Antipsychotic Drug Treatment in Schizophrenia Patients With or Without Metabolic Syndrome

**DOI:** 10.3389/fpsyt.2019.00803

**Published:** 2019-11-01

**Authors:** Elena G. Kornetova, Alexander N. Kornetov, Irina A. Mednova, Viktoria V. Dubrovskaya, Anastasia S. Boiko, Nikolay A. Bokhan, Anton J. M. Loonen, Svetlana A. Ivanova

**Affiliations:** ^1^Mental Health Research Institute, Tomsk National Research Medical Center of the Russian Academy of Sciences, Tomsk, Russia; ^2^Hospital, Siberian State Medical University, Tomsk, Russia; ^3^Department of Fundamental Psychology and Behavioral Medicine, Siberian State Medical University, Tomsk, Russia; ^4^Department of Psychiatry, Addictology and Psychotherapy, Siberian State Medical University, Tomsk, Russia; ^5^Department of Psychotherapy and Psychological Counseling, National Research Tomsk State University, Tomsk, Russia; ^6^Groningen Research Institute of Pharmacy, PharmacoTherapy, Epidemiology and Economics, University of Groningen, Groningen, Netherlands; ^7^Policy Office for Quality and Innovation of Care (BZI), GGZ Westelijk Noord-Brabant, Halsteren, Netherlands; ^8^School of Non-Destructive Testing and Security, Division for Control and Diagnostics, National Research Tomsk Polytechnic University, Tomsk, Russia

**Keywords:** schizophrenia, metabolic syndrome, atypical antipsychotics, body fat, metabolic parameters, insulin resistance

## Abstract

**Background:** Metabolic syndrome (MetS) is a common problem in schizophrenia patients and associated with increased mortality due to cardiovascular disease. Second-generation antipsychotics (SGAs) play an important role in facilitating MetS.

**Objective:** The study aimed to assess weight changes and alterations of indicators of body fat composition and lipid-glucose metabolism induced by reinitiating atypical antipsychotics in patients with schizophrenia when with or without MetS.

**Methods:** After giving informed consent, newly admitted patients with a clinical diagnosis of schizophrenia (ICD-10: F20) and an age between 18 and 55 years were included. MetS was diagnosed according to International Diabetes Federation (IDF) criteria. At entry and after 6 weeks of treatment, anthropometry and biochemical analysis were carried out. Total and visceral fats were measured with the use of non-invasive bioimpedance analysis and subcutaneous fat with calculation of total adipose tissue with the use of caliperometry. Based on biochemical assessments low density (LDL) and very low-density lipoproteins (VLDL), atherogenic index and Homeostatic Model Assessment of Insulin Resistance (IR-HOMA) were calculated. Statistical analysis was conducted using Wilcoxon signed-rank test, Mann-Whitney U-test, and chi-squared test. Differences were considered statistically significant at *p* < 0.05.

**Results:** A total of 114 patients (59M/55F) with schizophrenia were examined; they were divided into two groups with (n = 43; 37.7%) and without (n = 71; 62.3%) MetS. After a 6-week SGA treatment, only the total fat fold, waist circumference, triglyceride level, and atherogenic index underwent statistically significant changes in patients with MetS. In those without MetS, statistically significant changes across all fat indicators were noted. Also, a significant increase in blood glucose and HOMA-IR parameters, triglyceride, and VLDL levels and atherogenic index was observed in this group.

**Discussion:** The study illustrates the benefits of estimating both anthropometric and biochemical parameters shortly after (re)installing treatment of schizophrenia in order to minimize the risk of MetS development.

## Introduction

Schizophrenia is a chronic mental disorder with a 12-month prevalence of 3.70 per 1,000 or 0.33% and a median life time prevalence of 6.35 per 1,000 or 0.48% according to Moreno-Küstner et al. (1) or Simeone et al. (2), respectively. Schizophrenia is complicated by significant social and physical disabilities resulting in a number of years of potential life lost, which was assessed to amount to an average of 14.5 (95% CI 11.2–17.8) and is accompanied by an average life expectancy that was calculated to be 64.7 years (95% CI 61.1–68.3) (3). A primary cause of this excess of mortality may be the high incidence of cardiovascular disease, which was estimated to amount to 1.53 (CI95% 1.27–1.86) for schizophrenia in comparison with the reference group according to a meta-analysis involving over 3.5 million participants combining 13 studies (4). A major contribution to the risk of developing cardiovascular disease may be the existence of metabolic syndrome (MetS). The relative risk for cardiovascular disease over 5–10 years amounted approximately twofold in the general population with a diagnosis of MetS, and the risk for type 2 diabetes was even fivefold increased (5). MetS has an overall rate of 32.5% (95% CI 30.1%–35.0%) in patients with schizophrenia (6). Older age, and particularly illness durations, had a strong influence on the prevalence of MetS (6). This is probably partly, but not exclusively, related to the usage of psychotropic drugs such as clozapine and second-generation antipsychotics (SGAs). Although the prevalence of MetS is not significantly different in first-episode, antipsychotic-naïve patients in comparison to healthy controls (7, 8), abnormalities of glucose homeostasis already exist (9). This may be related to a shared underlying pathophysiology between schizophrenia and diabetes mellitus involving inflammation (10, 11). The same is probably true for the relationship between schizophrenia and dyslipidemia [in MetS defined as a decreased high density lipoprotein (HDL)-cholesterol and elevated triglycerides] (12). Over 20% of the treatment-naïve (prodromal or first-episode) schizophrenia patients have a lowered HDL-cholesterol level and a glucose dysregulation/insulin resistance (7, 13).

Although dyslipidemia is already more prevalent in schizophrenia patients before or during the debut of their disorder, treatment with antipsychotic drugs (particularly SGAs) has profound metabolic effects (14, 15, 16). Vancampfort et al. (8) found that schizophrenia patients with multiple episodes had a significantly (*p* < 0.001) higher prevalence of abdominal obesity, low high-density lipoprotein cholesterol, hypertriglyceridemia, and MetS compared to first-episode and drug-naïve patients. A typical component of MetS in patients with schizophrenia is central obesity as reflected by increased waist circumference, which is due to expansion of the amount of abdominal fat (5, 11). This abdominal type obesity consists of two components: visceral and subcutaneous. Especially visceral type obesity correlates with an unfavorable course of MetS (17, 18). In patients with schizophrenia, visceral fat has increased relative to properly matched healthy controls (19). This is particularly true for pericardial and, to a lesser extent, to intra-abdominal obesity (20).

Comparison of the weight gain after the initiation of antipsychotic treatment showed that all first- and second-generation antipsychotics may cause this phenomenon; however, the amount varies largely between different substances: clozapine and olanzapine seem to be the two compounds with the highest risk, a mid-risk group is composed of amisulpride, asenapine, iloperidone, paliperidone, quetiapine, risperidone, and sertindole and compounds with the lowest risk are aripiprazole, lurasidone, and ziprasidone (16). Weight gain mostly occurs during the first weeks of treatment, but does not seem to plateau in longer treatment phases (16). Nevertheless, an important difference is expected when patients with or without MetS start with an SGA. Whether or not other components of MetS (specifically HDL) react in parallel to the weight change is hitherto unknown. Until now, insufficient attention has moreover been paid to changes in the fatty component of body composition and biochemical parameters during the treatment of patients with schizophrenia in routine psychiatric practice.

The aim of our study is to assess changes of weight, body fat composition, and biochemical parameters of patients with schizophrenia receiving atypical antipsychotics, between groups with and without MetS.

## Materials and Methods

### Participants

This study was carried out in accordance with the Code of Ethics of the World Medical Association (Declaration of Helsinki 1975, revised in Fortaleza, Brazil, 2013), established for experiments involving humans. The study was approved (protocol N187, 24.04.2018) by the Local Bioethics Committee of the Mental Health Research Institute, Tomsk, Russia. Informed consent was obtained from all participants after proper explanation was given. The inclusion criteria were a clinical diagnosis of schizophrenia, according to the International Statistical Classification of Diseases and Related Health Problems, 10th Revision (ICD-10: F20) and an age between 18 and 55 years. Symptoms severity assessment was carried out with the Positive and Negative Syndrome Scale (21) at the beginning of the observation. Most patients received SGAs in maintenance dosages before admission to the hospital [olanzapine, 30 patients (26.32%), quetiapine, 34 (29.82%), risperidone, 43 (37.72%), or clozapine, 7 (6.14%)]. They were often non-adherent though and were hospitalized due to exacerbation of symptoms of schizophrenia. All patients continued to receive these SGAs in standard therapeutic dosages after recruiting to the study.

### Definition of Metabolic Syndrome

Patients were divided into two groups, either with or without MetS, according to the criteria of the International Diabetes Federation (IDF) (5, 17, 22). These criteria demand that MetS is diagnosed in a patient with central obesity (waist circumference more than 94 cm in men and more than 80 cm in women) and the presence of any two of the following four signs:

- the concentration of triglycerides in serum is higher than 1.7 mmol/L (150 mg/dl) or lipid-lowering therapy is carried out;
- the concentration of high-density lipoprotein in serum is below 1.03 mmol/L (40 mg/dl) in men and 1.29 mmol/L (50 mg/dl) in women;
- the arterial blood pressure level is systolic above 130 mmHg or diastolic above 85 mmHg (or with treatment of previously diagnosed hypertension);
- serum glucose concentration is greater than 5.6 mmol/L (100 mg/dl) (or previously diagnosed type 2 diabetes).

### Assessment of Body Fat Composition

Waist circumference was measured with a measuring tape. The body fat percentage, visceral fat level, body weight, and body mass index were determined through the non-invasive bioimpedance analysis with an “Omron BF508” scale and body composition monitor. Indicators of subcutaneous fat (total fat fold) were determined by an electronic caliper. The total fat fold consists of the sum of the fat folds in the shoulder, back, abdomen, and lower leg. These anthropometric measurements fully covered the composition of the entire body fat compartment, including indicators of subcutaneous as well as visceral fat. The indicators were measured twice: upon admission to the clinic and after 6 weeks of therapy.

### Blood Sampling

Blood samples were drawn after 12 h overnight fasting by antecubital venipuncture into tubes (BD Vacutainer) with a clot activator (CAT) in the first days of hospitalization and after a 6-week treatment with SGAs. To isolate serum samples, the tubes were centrifuged for 30 min at 2,000 *g* at 4°C. The serum was stored at −20°C (or –80°C), until analysis.

### Biochemical Parameters

Concentration of total cholesterol (TC), high density lipoproteins (HDL), triglycerides (TG), and glucose in blood serum was determined by colorimetric enzymatic methods applying standard commercial kits (Cormay, Poland). Concentrations of low density (LDL) and very low-density lipoproteins (VLDL) were calculated from the formula of W.T. Friedewald et al. (23). Atherogenic index was calculated from the formula offered by A.N. Klimov (24). Concentration of insulin was determined with use of immune-enzyme analysis (Vector Best, Russia). Insulin resistance was assessed by calculating HOMA-IR using the formula of D.R. Matthews et al. (25).

### Statistical Analysis

Statistical analyses were performed using the SPSS software for Windows, version 20.0. The normal distribution of the data was tested with the Shapiro-Wilk test. Between-group differences were evaluated using the Wilcoxon signed-rank test for related samples with non-normal distribution, and the Mann-Whitney U-test for independent samples with non-normal distribution. The categorical variables were analyzed using the chi-square (*Χ^2^*) test. Descriptive statistics were showed as median with 25% and 75% quartiles (Me [Q1; Q3]). P-values less than 0.05 were considered significant.

## Results

We recruited 114 newly admitted patients with schizophrenia from the Mental Health Research Institute Inpatient Department (**Table 1**); these patients were divided into two groups with (n = 43; 37.7%) and without (n = 71; 62.3%) MetS. No differences were observed between treatment upon admission, but significant differences existed in age (*p* = 0.0005) and in duration of illness (*p* = 0.0003).

**Table 1:** Characteristics of patients depending on the presence or absence of MetS.

Characteristics	Patients with MetS (n = 43)	Patients without MetS (n = 71)	p-value
Age, Me [Q1; Q3] years	39 [30; 52]	32 [27; 37]	0.0005*
Sex [male, n (%)/female, n (%)]	27 (62.8)/16 (37.2)	32 (45.1)/39 (54.9)	0.067
Schizophrenia onset age, Me [Q1; Q3]	23 [19; 29]	22 [19; 26]	0.3162
Duration of disease, Me [Q1; Q3] years	16 [10; 22]	9 [4; 15]	0.0003*
Chlorpromazine equivalent doses, Me [Q1; Q3]	300 [212.5; 600]	400 [200; 600]	0.4657
PANSS, Me [Q1; Q3] total score	100 [87; 108]	94 [81; 106]	0.1299

*p < 0.05—statistically significant difference. Comparisons between groups were performed using chi-squared test for sex and Mann-Whitney U-test for the rest of the variables.

No statistically significant differences existed with respect to changes of indicators for the total body fat percentage (*p* = 0.3090), visceral fat level (*p* = 0.5408), body weight (*p* = 0.1148), and body mass index (*p* = 0.2579) during a 6-week re-installed treatment of patients with MetS. Only total fat fold and waist circumference underwent statistically significant change (*p* = 0.0067 and *p* = 0.0342, respectively). Statistically significant changes, however, across all indicators in the group without MetS were noted: total body fat percentage (*p* < 0.0001), visceral fat level (*p* = 0.0010), total fat fold (*p* < 0.0001), waist circumference (*p* = 0.0002), body weight (*p* < 0.0001), and body mass index (*p* < 0.0001) (**Table 2**).

**Table 2:** Body fat composition before and after re-installing SGAs in patients with schizophrenia during a 6-week treatment depending on the presence or absence of MetS [Me (Q1; Q3)].

Time-point for the assessment	Patients with MetS (n = 43)	p-value	Patients without MetS (n = 71)	p-value
Total body fat percentage				
At hospitalization	36.6 [30; 47.6]	0.3090	31.6 [22.3; 40.3]	<0.0001*
After a 6-week treatment	36.6 [31.6; 47.7]		32.2 [24.8; 42]	
Visceral fat level				
At hospitalization	10 [9; 14]	0.5408	6 [4; 8]	0.0010*
After a 6-week treatment	11 [9; 14]		6 [4; 9]	
Total fat fold				
At hospitalization, mm	116 [93; 141]	0.0067*	84 [62; 98]	<0.0001*
After a 6-week treatment, mm	121 [93; 143]		88 [63; 108]	
Waist circumference				
At hospitalization, cm	106 [99; 114]	0.0342*	86 [77; 96]	0.0002*
After a 6-week treatment, cm	108 [98; 116]		85 [78; 97]	
Body weight				
At hospitalization, kg	95.6 [87.1; 108.3]	0.1148	71.9 [62; 84.1]	<0.0001*
After a 6-week treatment, kg	97.3 [88; 108.7]		74.0 [64; 85.7]	
Body mass index				
At hospitalization	31.55 [27; 36]	0.2579	24.7 [22.3; 28.4]	<0.0001*
After a 6-week treatment	31,95 [28.7; 35.7]		26.0 [22.7; 29.6]	

*p < 0.05—statistically significant difference. Comparisons between groups were performed using the Wilcoxon signed-rank test.

We observed no significant differences with respect to parameters of glucose metabolism in patients with schizophrenia with MetS after a 6-week re-installed treatment. Both TC level and atherogenic index had significantly increased in this patient group (*p* = 0.021 and *p* = 0.029, respectively). In contrast, we found a significant increase of both glucose metabolism [glucose (*p* < 0.001), HOMA-IR (*p* = 0.023)] and lipid metabolism parameters [triglycerides (*p* < 0.001), VLDL (*p* < 0.001), and atherogenic index (*p* = 0.042)] in patients without MetS at entry after a 6-week re-installed treatment (**Table 3**).

**Table 3:** Glucose and lipid metabolism dynamics in patients with schizophrenia during a 6-week treatment depending on the presence or absence of MetS [Me (Q1; Q3)].

Time-point for the assessment	Patients with MetS (n = 43)	p-value	Patients without MetS (n = 71)	p-value
*Glucose metabolism*				
Glucose				
At hospitalization, mmol/l	5.15 [4.60; 5.60]	0.345	4.95 [4.75; 5.10]	<0.001*
After a 6-week treatment, mmol/l	5.20 [4.70; 5.90]		5.35 [4.80; 5.60]	
Insulin				
At hospitalization, µIU/ml	3.75 [2.02; 8.59]	0.482	0.65 [0.12; 2.79]	0.079
After a 6-week treatment, µIU/ml	5.54 [2.21; 9. 75]		1.50 [0.58; 4.57]	
HOMA-IR				
At hospitalization	0.17 [0. 28; 0.43]	0.053	0.17 [0. 28; 0.43]	0.023*
After a 6-week treatment	0.32 [0.12; 0. 94]		0.32 [0.12; 0. 94]	
*Lipid metabolism*				
Total cholesterol				
At hospitalization, mmol/l	4.63 [4.09; 5.10]	0.021*	4.64 [3.83; 5.15]	0.083
After a 6-week treatment, mmol/l	4.79 [3.92; 5.49]		4.63 [3.98; 5.21]	
Triglycerides				
At hospitalization, mmol/l	2.05 [1.75; 1.55]	0.073	1.05 [0.71; 1.25]	<0.001*
After a 6-week treatment, mmol/l	2.45 [1.85; 2.72]		1.52 [1.22; 2.00]	
High-density lipoproteins				
At hospitalization, mmol/l	1.00 [0.80; 1.10]	0.706	1.23 [1.05; 1.40]	0.336
After a 6-week treatment, mmol/l	0.85 [0.60-1.35]		1.00 [0.80-1.40]	
Low-density lipoproteins				
At hospitalization, mmol/l	2.80 [1.95; 3.20]	0.242	2.90 [2.12; 3.65]	0.466
After a 6-week treatment, mmol/l	2.57 [1.65; 3.37]		2.72 [2.34; 3.40]	
Very low-density lipoproteins				
At hospitalization, mmol/l	0.93 [0.80; 1.16]	0.073	0.48 [0.32; 0.57]	<0.001*
After a 6-week treatment, mmol/l	1.1 [0.84; 1.24]		0.69 [0.56; 0.91]	
Atherogenic index				
At hospitalization	3.74 [2.94; 5. 51]	0.029*	2.82 [1.92; 3.54]	0.042*
After a 6-week treatment	3.99 [2.32; 7.65]		3.69 [2.31; 5.39]	

*p < 0.05—statistically significant difference. HOMA-IR: homeostasis model assessment of insulin resistance. Comparisons between groups were performed using the Wilcoxon signed-rank test.

In addition, changes in body fat composition and biochemical parameters, depending on the drug taken, were analyzed, with the exception of seven patients taking clozapine due to sample size.

Patients with MetS treated with olanzapine, quetiapine, and risperidone did not have statistically significant changes in the indicators of fat composition after a 6-week treatment (**Tables 4**, **6**, and **8**); on the contrary, in patients without MetS who took olanzapine and quetiapine, all indicators of fat composition increased significantly (**Tables 4** and **6**). In patients without MetS who used risperidone, the following fat parameters were significantly increased (**Table 8**): total body fat percentage (*p* = 0.020), waist circumference (*p* = 0.020), body weight (*p* = 0.002), and body mass index (*p* = 0.001)

**Table 4:** Body fat composition before and after re-installing SGAs in patients with schizophrenia during a 6-week treatment with olanzapine depending on the presence or absence of MetS [Me (Q1; Q3)].

Time-point for the assessment	Patients with MetS (n = 10)	p-value	Patients without MetS (n = 20)	p-value
Total body fat percentage				
At hospitalization	46.5 [36; 53.6]	0.944	31.2 [22.6; 30.0]	0.005*
After a 6-week treatment	45.5 [35.9; 53.7]		35.1 [25.4; 44.1]	
Visceral fat level				
At hospitalization	13 [9; 18]	0.963	7 [4; 8]	0.034*
After a 6-week treatment	13 [10; 17]		8 [5; 9]	
Total fat fold				
At hospitalization, mm	119 [102; 146]	0.341	84 [68; 116]	<0.001*
After a 6-week treatment, mm	124 [104; 146]		85 [70; 118]	
Waist circumference				
At hospitalization, cm	114 [103; 125]	0.105	87 [80; 95]	0.020*
After a 6-week treatment, cm	115 [105; 125]		88 [80; 95]	
Body weight				
At hospitalization, kg	103.1 [95.0; 119.1]	0.306	73.7 [62.3; 87.9]	0.029*
After a 6-week treatment, kg	103.3 [96.4; 117.6]		74.2 [64; 89.5]	
Body mass index				
At hospitalization	36.1 [34.5; 37.8]	0.889	25.0 [22.6; 30.0]	0.023*
After a 6-week treatment	36.0 [34.4; 37.8]		26.7 [23.6; 34.1]	

*p < 0.05—statistically significant difference. Comparisons between groups were performed using the Wilcoxon signed-rank test.

Also, patients with MetS treated with olanzapine, quetiapine, and risperidone did not have statistically significant changes in the biochemical parameters after a 6-week treatment (**Tables 5**, **7**, and **9**). In patients without MetS who used olanzapine, the following biochemical indicators were significantly increased (**Table 5**): triglycerides (*p* = 0.030), high-density lipoproteins (*p* = 0.027), very low-density lipoproteins (*p* = 0.030), and atherogenic index (*p* = 0.040). In patients without MetS who used quetiapine, the following biochemical parameters were significantly increased (**Table 7**): glucose (*p* < 0.001), HOMA-IR (*p* = 0.050), triglycerides (*p* = 0.009), and very low-density lipoproteins (*p* = 0.009). In patients without MetS who used risperidone only, triglycerides (*p* = 0.001) and very low-density lipoproteins (*p* = 0.001) were significantly increased (**Table 9**).

**Table 5:** Glucose and lipid metabolism dynamics in patients with schizophrenia during a 6-week treatment with olanzapine depending on the presence or absence of MetS [Me (Q1; Q3)].

Time-point for the assessment	Patients with MetS (n = 10)	p-value	Patients without MetS (n = 20)	p-value
*Glucose metabolism*				
Glucose				
At hospitalization, mmol/l	4.90 [4.60; 5.60]	0.097	4.90 [4.80; 5.30]	0.380
After a 6-week treatment, mmol/l	5.70 [5.00; 6.00]		5.10 [4.30; 5.50]	
Insulin				
At hospitalization, µIU/ml	8.01 [2.95; 12.33]	0.500	1.91 [1.00; 10.34]	0.594
After a 6-week treatment, µIU/ml	8.49 [3.71; 14. 07]		6.42 [0.63; 11.63]	
HOMA-IR				
At hospitalization	1.77 [0. 71; 3.19]	0.500	0.40 [0. 21; 2.27]	0.131
After a 6-week treatment	1.74 [1.05; 3. 51]		1.83 [0.13; 3. 15]	
*Lipid metabolism*				
Total cholesterol				
At hospitalization, mmol/l	4.51 [4.13; 5.59]	0.260	4.72 [4.13; 5.37]	0.232
After a 6-week treatment, mmol/l	5.38 [3.98; 6.36]		4.95 [4.41; 6.15]	
Triglycerides				
At hospitalization, mmol/l	2.03 [1.75; 2.80]	0.192	1.01 [0.80; 1.50]	0.030*
After a 6-week treatment, mmol/l	2.87 [2.00; 4.40]		1.40 [0.95; 1.95]	
High-density lipoproteins				
At hospitalization, mmol/l	0.90 [0.70; 1.03]	0.634	1.16 [0.93; 1.30]	0.027*
After a 6-week treatment, mmol/l	0.90 [0.77; 1.11]		0.99 [0.86; 1.26]	
Low-density lipoproteins				
At hospitalization, mmol/l	2.64 [2.05; 3.90]	0.515	2.93 [2.50; 3.81]	0.191
After a 6-week treatment, mmol/l	2.68 [2.10; 3.74]		3.20 [2.43; 4.57]	
Very low-density lipoproteins				
At hospitalization, mmol/l	0.92 [0.79; 1.28]	0.192	0.46 [0.36; 0.68]	0.030*
After a 6-week treatment, mmol/l	1.30 [0.91; 2.00]		0.63 [0.43; 0.89]	
Atherogenic index				
At hospitalization	5.19 [3.23; 7.06]	0.859	2.92 [2.52; 3.82]	0.040*
After a 6-week treatment	5.15 [2.88; 5.89]		3.94 [2.48; 5.75]	

*p < 0.05—statistically significant difference. HOMA-IR, homeostasis model assessment of insulin resistance. Comparisons between groups were performed using the Wilcoxon signed-rank test.

**Table 6:** Body fat composition before and after re-installing SGAs in patients with schizophrenia during a 6-week treatment with quetiapine depending on the presence or absence of MetS [Me (Q1; Q3)].

Time-point for the assessment	Patients with MetS (n = 13)	p-value	Patients without MetS (n = 21)	p-value
Total body fat percentage				
At hospitalization	41.2 [30; 47.2]	0.139	31.6 [24.6; 42.7]	0.003*
After a 6-week treatment	41.3 [30.6; 47.9]		32.2 [26.7; 42.1]	
Visceral fat level				
At hospitalization	10 [9; 14]	0.987	7 [4; 10]	0.020*
After a 6-week treatment	10 [9; 13]		7 [5; 11]	
Total fat fold				
At hospitalization, mm	122 [92; 150]	0.074	91 [67; 107]	0.007*
After a 6-week treatment, mm	127 [95; 154]		91 [73; 111]	
Waist circumference				
At hospitalization, cm	104 [101; 110]	0.233	88 [79; 96]	0.021*
After a 6-week treatment, cm	107 [100; 111]		89 [79; 97]	
Body weight				
At hospitalization, kg	97.0 [85.6; 111.7]	0.346	71.3 [61.1; 84.8]	<0.001*
After a 6-week treatment, kg	99.6 [84.4; 111.7]		74.6 [61.3; 85.8]	
Body mass index				
At hospitalization	32.2 [26.9; 37.6]	0.173	26.1 [22.1; 29.8]	<0.001*
After a 6-week treatment	32.6 [29.1; 38.4]		26.8 [22.8; 29.8]	

*p < 0.05—statistically significant difference. Comparisons between groups were performed using the Wilcoxon signed-rank test.

**Table 7:** Glucose and lipid metabolism dynamics in patients with schizophrenia during a 6-week treatment with quetiapine depending on the presence or absence of MetS [Me (Q1; Q3)].

Time-point for the assessment	Patients with MetS (n = 13)	p-value	Patients without MetS (n = 21)	p-value
*Glucose metabolism*				
Glucose				
At hospitalization, mmol/l	5.70 [4.60; 6.20]	0.859	5.10 [4.30; 5.30]	<0.001*
After a 6-week treatment, mmol/l	5.70 [5.00; 6.00]		5.30 [5.10; 5.80]	
Insulin				
At hospitalization, µIU/ml	4.03 [0.97; 8.68]	0.441	1.04 [0.64; 4.99]	0.101
After a 6-week treatment, µIU/ml	5.93 [0.58; 13. 76]		2.29 [0.94; 4.49]	
HOMA-IR				
At hospitalization	1.12 [0.20; 2.16]	0.374	0.23 [0. 12; 1.01]	0.050*
After a 6-week treatment	1.53 [0.16; 3.67]		0.59 [0.27; 1. 09]	
*Lipid metabolism*				
Total cholesterol				
At hospitalization, mmol/l	4.11 [3.50; 5.11]	0.136	4.59 [4.07; 5.70]	0.848
After a 6-week treatment, mmol/l	4.78 [4.06; 5.74]		4.70 [4.00; 5.30]	
Triglycerides				
At hospitalization, mmol/l	1.80 [1.60; 2.75]	0.480	1.30 [0.81; 1.50]	0.009*
After a 6-week treatment, mmol/l	2.32 [1.73; 2.73]		1.50 [1.25; 1.95]	
High-density lipoproteins				
At hospitalization, mmol/l	0.90 [0.75; 1.15]	0.575	1.20 [1.10; 1.45]	0.574
After a 6-week treatment, mmol/l	1.05 [0.68; 1.52]		1.30 [0.98; 1.40]	
Low-density lipoproteins				
At hospitalization, mmol/l	2.48 [1.87; 3.10]	0.583	2.70 [2.25; 3.90]	0.876
After a 6-week treatment, mmol/l	2.53 [1.88; 3.42]		2.52 [2.20; 3.53]	
Very low-density lipoproteins				
At hospitalization, mmol/l	0.82 [0.73; 1.25]	0.480	0.59 [0.37; 0.68]	0.009*
After a 6-week treatment, mmol/l	1.05 [0.78; 1.25]		0.68 [0.57; 0.89]	
Atherogenic index				
At hospitalization	3.57 [2.82; 4.39]	0.583	2.70 [2.19; 4.23]	0.476
After a 6-week treatment	3.65 [1.95; 5.93]		2.93 [2.24; 4.12]	

*p < 0.05—statistically significant difference. HOMA-IR: homeostasis model assessment of insulin resistance. Comparisons between groups were performed using the Wilcoxon signed-rank test.

**Table 8:** Body fat composition before and after re-installing SGAs in patients with schizophrenia during a 6-week treatment with risperidone depending on the presence or absence of MetS [Me (Q1; Q3)].

Time-point for the assessment	Patients with MetS (n = 15)	p-value	Patients without MetS (n = 28)	p-value
Total body fat percentage				
At hospitalization	33.9 [28.9; 41.9]	0.889	27.1 [16.7; 35.7]	0.020*
After a 6-week treatment	33.9 [28.4; 43.2]		29.2 [16.7; 36.1]	
Visceral fat level				
At hospitalization	10 [8; 12]	0.257	5 [4; 7]	0.068
After a 6-week treatment	10 [8; 12]		5 [3; 7]	
Total fat fold				
At hospitalization, mm	98 [82; 118]	0.582	74 [45; 88]	0.066
After a 6-week treatment, mm	101 [81; 121]		70 [49; 90]	
Waist circumference				
At hospitalization, cm	104 [96; 113]	0.538	80 [74; 90]	0.020*
After a 6-week treatment, cm	105 [96; 113]		82 [74; 94]	
Body weight				
At hospitalization, kg	91.0 [78.0; 99.1]	0.649	71.9 [61; 80.3]	0.002*
After a 6-week treatment, kg	92.9 [78.2; 100.1]		71.9 [63.4; 81.9]	
Body mass index				
At hospitalization	29.9 [26.5; 31.2]	0.723	23.2 [21.6; 25.8]	0.001*
After a 6-week treatment	30.2 [26.7; 31.8]		23.6 [21.8; 27.5]	

*p < 0.05—statistically significant difference. Comparisons between groups were performed using the Wilcoxon signed-rank test.

**Table 9:** Glucose and lipid metabolism dynamics in patients with schizophrenia during a 6-week treatment with risperidone depending on the presence or absence of MetS [Me (Q1; Q3)].

Time-point for the assessment	Patients with MetS (n = 15)	p-value	Patients without MetS (n = 28)	p-value
*Glucose metabolism*				
Glucose				
At hospitalization, mmol/l	4.90 [4.70; 5.40]	0.888	4.90 [4.30; 5.10]	0.052
After a 6-week treatment, mmol/l	5.10 [4.70; 5.50]		5.00 [4.60; 5.40]	
Insulin				
At hospitalization, µIU/ml	1.70 [0.10; 7.91]	0.213	1.05 [0.12; 4.31]	0.535
After a 6-week treatment, µIU/ml	2.52 [0.95; 8.80]		1.47 [0.49; 2.84]	
HOMA-IR				
At hospitalization	0.34 [0.02; 1.68]	0.182	0.22 [0.03; 0.98]	0.642
After a 6-week treatment	0.51 [0.22; 1.97]		0.33 [0.10; 0.61]	
*Lipid metabolism*				
Total cholesterol				
At hospitalization, mmol/l	5.01 [3.79; 5.73]	0.281	4.00 [3.49; 4.71]	0.113
After a 6-week treatment, mmol/l	5.19 [4.31; 5.52]		4.33 [3.47; 5.60]	
Triglycerides				
At hospitalization, mmol/l	2.00 [1.30; 2.10]	0.167	1.00 [0.70; 1.40]	<0.001*
After a 6-week treatment, mmol/l	2.15 [1.59; 2.50]		1.50 [0.92; 2.00]	
High-density lipoproteins				
At hospitalization, mmol/l	1.00 [0.80; 1.20]	0.456	1.10 [0.90; 1.50]	0.589
After a 6-week treatment, mmol/l	0.80 [0.40; 1.10]		1.19 [0.95; 1.50]	
Low-density lipoproteins				
At hospitalization, mmol/l	3.00 [1.90; 3.80]	0.496	2.59 [2.00; 2.87]	0.971
After a 6-week treatment, mmol/l	3.45 [2.51; 4.12]		2.55 [1.72; 3.15]	
Very low-density lipoproteins				
At hospitalization, mmol/l	0.91 [0.59; 0.95]	0.167	0.50 [0.32; 0.64]	<0.001*
After a 6-week treatment, mmol/l	0.98 [0.72; 1.14]		0.73 [0.45; 0.91]	
Atherogenic index				
At hospitalization	3.80 [3.22; 4.57]	0.088	2.70 [1.88; 3.73]	0.548
After a 6-week treatment	5.56 [2.91; 11.95]		2.58 [2.21; 3.82]	

*p < 0.05—statistically significant difference. HOMA-IR, homeostasis model assessment of insulin resistance. Comparisons between groups were performed using the Wilcoxon signed-rank test.

## Discussion

In the present study, we observed that patients with MetS were significantly older than patients without MetS (**Table 1**). This result is consistent with the meta-analysis data of Mitchel et al. (6), which indicates an increase in the frequency of MetS in patients with schizophrenia over 38 years old. But according to this meta-analysis, the strongest influence on the rate of MetS is the duration of schizophrenia. This conclusion fully corresponds to our findings regarding the significantly longer disease duration and similar age of onset in the group of patients with in comparison to those without MetS.

The observation period with an assessment of body fat composition and biochemical parameters was 6 weeks. A short period of time for assessing changes in body fat composition and biochemical parameters was chosen due to the need to find ways to earlier assess the risk of developing MetS. Also, according to the “Meta-guidelines” for the management of patients with schizophrenia by Stahl et al. (26), the adequacy of the treatment trial must be assessed as a minimum of 3 weeks and a maximum of 6 weeks before making a major change to the treatment regimen.

The variables indicating the body fat composition show significant increase during a 6-week reinstated SGA treatment concerning all indicators in the group without MetS. This contrasts the results in patients with MetS, which only showed significant increase in total fat fold and waist circumference. The other indicators in this group were relatively stable (**Table 2**). Thus, in patients with MetS, the changed body fat composition only reflected an increase in the amount of subcutaneous fat, while in the group of participants without MetS, both subcutaneous and visceral fat were raised. It has been suggested that visceral obesity is the primary determinant of insulin resistance representing the basic pathophysiological changes leading to MetS and, as a consequence, diabetes (27). A limitation of our study may be the presence of statistically significant age difference between groups with and without MetS (**Table 1**). However, the average age of patients in each group is in the same age cohort (20–39 years) for measuring the percentage of body fat according to data obtained in a study of different ethnic groups (28).

The results of a number of studies have suggested that antipsychotics contribute to altered glucose metabolism in psychiatric patients compared to the general population (29, 30, 31), which is consistent with our findings (**Table 3**). Nevertheless, certain reports describe that no significant relationship exists between glucose metabolism and SGA treatment (29, 32). It can be hypothesized that these contradictions are related to insufficient division of the applied antipsychotics into first- and second-generation drugs and the lack of diagnosing MetS in patients with schizophrenia.

The observed changes of the serum lipid parameters (**Table 3**) largely correspond to literature data, which are reported as primarily marked elevations of serum triglyceride levels (12, 33, 34). The change was significant in patients without MetS. It is suggested that hypertriglyceridemia may be caused by SGAs through stimulation of hepatic triglyceride production and secretion or through inhibition of lipoprotein lipase-mediated triglyceride hydrolysis. In addition, an indirect mechanism may contribute, associated with obesity and insulin resistance, *via* exerting unfavorable effects both on the central nervous system and on the biology of adipose tissue (35). The increase in the total cholesterol level was small but significant in patients with MetS, but non-existent in patients without MetS.

All SGAs used in this study had a comparable effect on changes in body fat composition and biochemical parameters (**Tables 4**–**9**), with the exception of risperidone, which did not significantly affect glucose metabolism (**Table 9**). This confirms the evidence that these SGAs have the highest risk or mid-risk of metabolic disturbances in patients receiving these drugs (16). Treatment with olanzapine, quetiapine, risperidone, and clozapine was not randomly selected, which limits the relevance of the study. But all recruited patients were already using these drugs before admission, reflecting existing clinical reality.

Based on our findings and on literature data, we want to suggest that altered glucose and lipid metabolism may play a more important role during the onset of MetS. Six-week treatment with SGAs leads to significant changes in biochemical parameters in patients without MetS. However, schizophrenia patients with already established MetS do not suffer from these side effects in a similar severity. The absence of a change in glucose and most of lipid parameters may be related to wearing off of the tendency to increase fasting lipids and glucose once after MetS has developed. Our data are consistent with the findings in a study of Smith et al. (36, 37), which observed no significant differences in fasting measures of lipids and glucose after 5 months of treatment with olanzapine or risperidone in chronic patients with schizophrenia. It should be noted that triglyceride levels at entry were much higher in patients with MetS than in those without this diagnosis. Apparently a further increase in these levels is less likely to occur. The atherogenic index is the only parameter that differs after 6 weeks of treatment both in patients with and without MetS, which indicates the need for calculating this parameter routinely when monitoring for disturbed lipid metabolism.

In most studies, the MetS problem in patients with schizophrenia is evaluated through calculating its prevalence or the prevalence of the individual MetS components. In our study, we demonstrated for the first time through monitoring specific parameters that even within a relatively short period of 6 weeks treatment with SGAs, several indices of body fat composition may increase. This may indicate the necessity of carefully monitoring SGA-treated patients at the beginning of their usage. Moreover, a number of MetS indicators are reversible with proper case management. Measurement of waist circumference, carried out in most studies for the diagnosis of abdominal obesity, provides only conditional ideas about its severity, and computer-assisted tomography is a rather expensive and not suitable for continuous monitoring. Hence, there exists room for improvement.

The method of non-invasive bioimpedance analysis used by the current study is low cost, is easily reproducible, and can be widely applied in routine psychiatric practice. This method yields objective data on the composition of the body fat compartment of patients with schizophrenia. Concomitant monitoring of biochemical lipid and glucose parameters provides a complete picture of the formation of MetS in SGA-taking patients.

## Data Availability Statement

The datasets generated for this study are available on request to the corresponding author.

## Ethics Statement

The studies involving human participants were reviewed and approved by Local Bioethics Committee of the Mental Health Research Institute, Tomsk, Russia, protocol N187, 24.04.2018. The patients/participants provided their written informed consent to participate in this study.

## Author Contributions

EK and SI designed and supervised the study. IM and VD collected the blood samples, anthropometrics, and clinical information. AB measured the biochemical data. AK and IM carried out the statistical analysis. EK, AK, and IM wrote the first draft of the manuscript. SI, AL, and NB commented on this draft and contributed to the final manuscript.

## Funding

This work was supported by the Russian Science Foundation, grant # 18-15-00011.

## Conflict of Interest

The authors declare that this research was conducted in the absence of any commercial or financial relationships that could be construed as a potential conflict of interest.
